# Bacterial adenine cross-feeding stems from a purine salvage bottleneck

**DOI:** 10.1093/ismejo/wrae034

**Published:** 2024-03-07

**Authors:** Ying-Chih Chuang, Nicholas W Haas, Robert Pepin, Megan G Behringer, Yasuhiro Oda, Breah LaSarre, Caroline S Harwood, James B McKinlay

**Affiliations:** Department of Biology, Indiana University, Bloomington, IN 47405, United States; Biochemistry Program, Indiana University, Bloomington, IN 47405, United States; Department of Biology, Indiana University, Bloomington, IN 47405, United States; Department of Chemistry, Indiana University, Bloomington, IN 47405, United States; Department of Biological Sciences, Vanderbilt University, Nashville, TN 37235, United States; Department of Microbiology, University of Washington, Seattle, WA 98195, United States; Department of Biology, Indiana University, Bloomington, IN 47405, United States; Department of Plant Pathology, Entomology, and Microbiology, Iowa State University, Ames, IA 50011, United States; Department of Microbiology, University of Washington, Seattle, WA 98195, United States; Department of Biology, Indiana University, Bloomington, IN 47405, United States

**Keywords:** cross-feeding, purine, adenine, microbial interactions, Rhodopseudomonas, palustris, purine salvage, mutualism, excretion, microbial physiology

## Abstract

Diverse ecosystems host microbial relationships that are stabilized by nutrient cross-feeding. Cross-feeding can involve metabolites that should hold value for the producer. Externalization of such communally valuable metabolites is often unexpected and difficult to predict. Previously, we discovered purine externalization by *Rhodopseudomonas palustris* by its ability to rescue an *Escherichia coli* purine auxotroph. Here we found that an *E. coli* purine auxotroph can stably coexist with *R. palustris* due to purine cross-feeding. We identified the cross-fed purine as adenine. Adenine was externalized by *R. palustris* under diverse growth conditions. Computational modeling suggested that adenine externalization occurs via diffusion across the cytoplasmic membrane. RNAseq analysis led us to hypothesize that adenine accumulation and externalization stem from a salvage pathway bottleneck at the enzyme encoded by *apt*. Ectopic expression of *apt* eliminated adenine externalization, supporting our hypothesis. A comparison of 49 *R. palustris* strains suggested that purine externalization is relatively common, with 16 strains exhibiting the trait. Purine externalization was correlated with the genomic orientation of *apt*, but *apt* orientation alone could not always explain purine externalization. Our results provide a mechanistic understanding of how a communally valuable metabolite can participate in cross-feeding. Our findings also highlight the challenge in identifying genetic signatures for metabolite externalization.

## Graphical Abstract

**Graphical Abstract:**
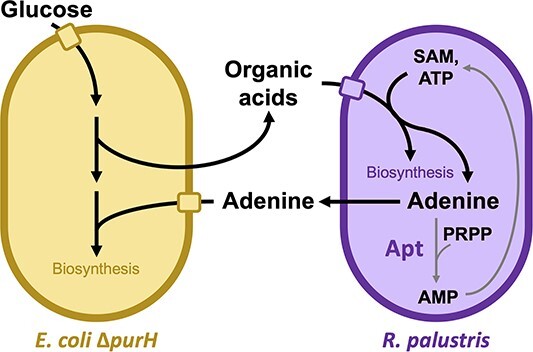

## Introduction

Cross-feeding between microbes is central to processes ranging from biogeochemical cycles to the human microbiome [1]. Although widespread, much remains unknown about the mechanisms underlying cross-feeding via metabolite externalization. Here we use externalization as a catch-all for any mode of moving metabolites out of a cell [2]. A particularly perplexing aspect of cross-feeding is the externalization of metabolites that hold value for both the recipient and the producer. Externalization of such communally valuable metabolites could pose a fitness cost for the producer, especially if the trait is exploited by non-reciprocating neighbors. Nonetheless, there are many examples of cross-feeding of communally valuable metabolites [1-5].

To study cross-feeding, many researchers use synthetic microbial communities, or cocultures. Cocultures preserve ecological aspects of interest while allowing control over environmental and genetic parameters [6]. Enforcing obligate cross-feeding of essential nutrients can ensure coexistence and reproducible outcomes. However, one cannot control or predict all the ways that microbes will interact. Understanding how microbes interact in synthetic communities is important to correctly interpret results from these research systems and to predict microbial interactions in nature [7, 8].

**Figure 1:**
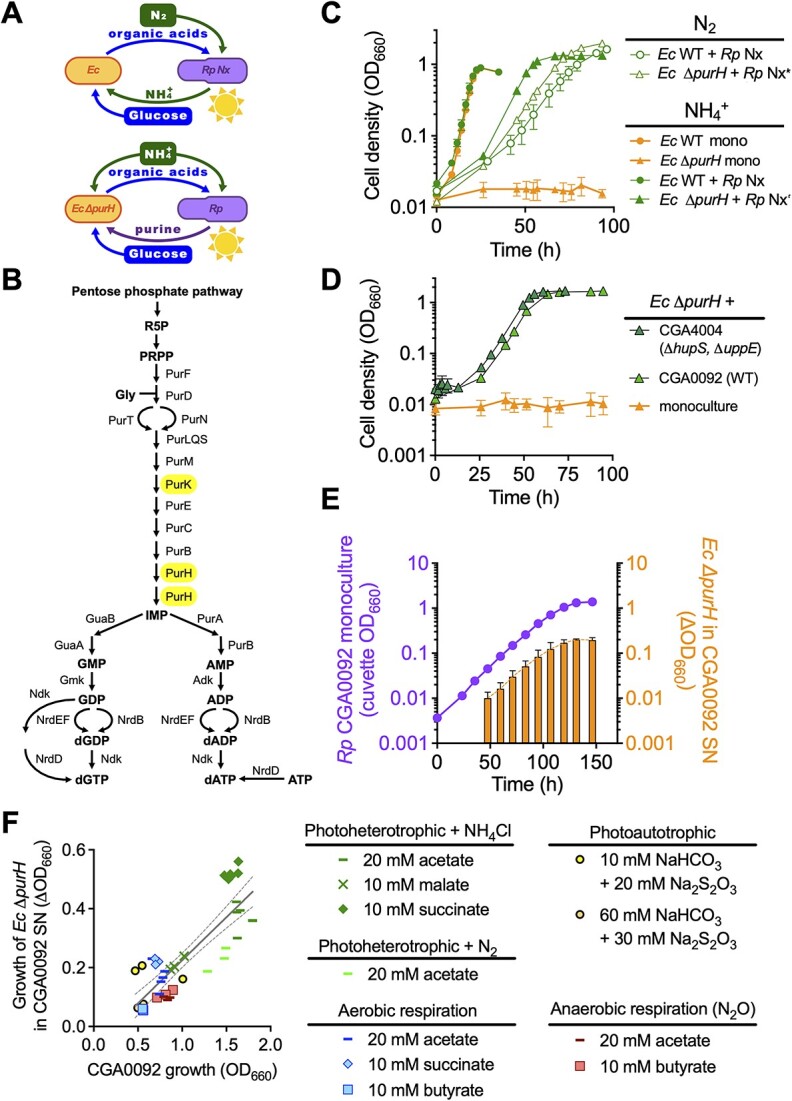
Wild-type *R. palustris* (*Rp*) CGA0092 supports *E. coli* (*Ec*) purine auxotroph growth across diverse growth conditions; (A) hypothesized critical cross-feeing interactions when N_2_ is the nitrogen source (top) or when *E. coli* is a purine auxotroph (bottom); (B) *E. coli* de-novo purine synthesis pathway; (C) growth curves for *E. coli* monocultures and cocultures with *R. palustris* NifA^*^ with either N_2_ or NH_4_Cl as the nitrogen source; (D) growth curves for *E. coli* Δ*purH* in coculture with *R. palustris* strains having a wild-type *nifA* gene; NH_4_Cl was the nitrogen source; (E) growth of *E. coli* Δ*purH* in supernatants from *R. palustris* CGA0092 monocultures; (C–E) error bars, SD; *n*=3; some error bars are smaller than the symbols; (F) growth of *E. coli* Δ*purH* in supernatants from stationary-phase CGA0092 monocultures grown under various growth conditions; each data point represents a single biological replicate. Linear regression (solid line) ± 95% confidence intervals (dashed lines) was applied to all samples across all conditions; SN, supernatant.

We previously designed a coculture where growth of fermentative *Escherichia coli* and *Rhodopseudomonas palustris* is dependent on the exchange of essential carbon and nitrogen in an anaerobic minimal medium (Fig. 1A, top) [9]. *R. palustris* is a metabolically versatile bacterium that can grow by either aerobic or anaerobic respiration, or by anoxygenic phototrophy with either inorganic carbon and electron sources (*R. palustris* cannot extract electrons from water and thus does not make O_2_) or with organic carbon [10, 11]. Only this latter photoheterotrophic lifestyle is available to *R. palustris* in the engineered coculture. *E. coli* ferments glucose to organic acids that provide essential carbon to *R. palustris*. *R. palustris* reciprocates by excreting ammonium (NH_4_^+^), derived from N_2_ gas. NH_4_^+^ excretion relies on constitutive nitrogenase activity (strain Nx, NifA^*^ mutation) [9].

In working with the above coculture, we uncovered an unanticipated interaction when we assessed the contribution of each *E. coli* gene to fitness in monoculture versus coculture using a transposon mutant library [12]. *E. coli* purine synthesis genes were dispensable in coculture but not in monoculture, suggesting that *R. palustris* externalized purine(s). Here, we characterize the molecular basis for purine externalization by *R. palustris* and the potential for purine cross-feeding in both synthetic and natural communities.

## Materials and methods

### Bacterial strains

Strains and 16S rRNA accession numbers are in Table S1. *R. palustris* CGA0092 differs from CGA009 by a single nucleotide polymorphism that affects pigmentation [10, 13]. *R. palustris* CGA4004 (Δ*hupS,* Δ*uppE*) is incapable of H_2_ oxidation and is deficient in cell aggregation [14]. *R. palustris* Nx (CGA4005; Δ*hupS,* ΔuppE, nifA^*^) excretes NH_4_^+^ under N_2_-fixing conditions [9]. *E. coli* MG1655 [15] deletion mutants were made via lambda Red recombination [16] using constructs amplified from Keio collection mutants [17]. Plasmids and primers are in Tables S2 and S3, respectively.

The adenosine phosphoribosyltransferase expression vector, pBBPgdh-apt, was generated using *E. coli*-mediated assembly [18] with *E. coli* NEB10β. Transformants were grown on lysogeny agar with 20 μg/ml gentamycin (Gm). Plasmids were identified by colony polymerase chain reaction (PCR) and verified by Sanger sequencing. Plasmids were transformed into CGA0092 by electroporation [19] and selected on photosynthetic medium agar [11] with 10 mM disodium succinate and 100 μg/ml Gm.

### Growth conditions

Anaerobic media were prepared by bubbling with N_2_, then sealing with rubber stoppers and aluminum crimps prior to autoclaving. Anaerobic cultures were grown in 10-ml volumes in 27-ml anaerobic test tubes or 60-ml volumes in 150-ml vials.

Single colonies were used to inoculate starter cultures. *R. palustris* starter cultures were grown anaerobically in minimal M9-derived coculture medium (MDC) [9] with 20 mM acetate and 10 mM NH_4_Cl. *E. coli* starter cultures were grown aerobically in lysogeny broth, with 30 μg/ml kanamycin when appropriate. To prepare *E. coli* for cocultures or bioassays, 0.2 ml of starter culture was centrifuged, and cell pellets were washed twice in 1 ml MDC. Cocultures were inoculated with 0.1 ml of *R. palustris* culture (diluted in MDC) and washed *E. coli* cells to an initial optical density (OD_660_) of ~0.003 each. Cocultures were grown anaerobically in MDC with 25 mM glucose, 10 mM NH_4_Cl, and 1% v/v cation solution (1 mM MgSO_4_ and 0.1 mM CaCl_2_), unless indicated otherwise. Photoautotrophic conditions used NaHCO_3_ and Na_2_S_2_O_3_ in place of organic carbon. Anaerobic respiration conditions used 0.1 mM NaNO_3_ and 100% N_2_O [11]. All cultures were incubated horizontally at 30°C with shaking at 150 rpm. Light was provided by a 45 W halogen bulb (430 lumens).

### Analytical procedures

Cell densities were measured via turbidity (OD_660_) using a Genesys 20 spectrophotometer (Thermo-Fisher). Glucose and fermentation products were measured using high-performance liquid chromatography (HPLC; Shimadzu) as described [20].

### Invasion-from-rare assays

Invasion-from-rare assays were used to test the invasion criterion for coexistence, where each population can increase when rare [21, 22]. Cocultures were started from various initial frequencies for a total initial cell density of ~10^6^ colony-forming units (CFUs) / ml. Initial *E. coli* Δ*purH* frequencies ranged from 0.008% to 99.898%. Samples were taken upon inoculation and after 5 days to determine frequencies. Change in frequency = (*E. coli* / (*E. coli* + *R. palustris*))_final_ – (*E. coli* / (*E. coli* + *R. palustris*))_initial_ [23].

### Metabolite extraction

*R. palustris* supernatants (1 ml) were spiked with internal standards of ^13^C_5_-adenosine (97%) and ^15^N_3_-dCMP (98%) (Cambridge Isotope Laboratories). Compounds were extracted with four volumes of 1:1 v/v acetonitrile/methanol at −20°C for 20 min before centrifugation at 18 400 x *g* at 4°C for 15 min. Supernatants were lyophilized and stored at −20°C.

Intracellular compounds were extracted as described [24]. Briefly, ~2 × 10^9^ cells were vacuum-filtered through a nylon membrane (0.45 μm). The membrane was transferred, cell-side down, into a Petri dish containing 2.5 ml of 40:40:20 v/v/v acetonitrile/methanol/water at −20°C and incubated at −20°C for 20 min. The solution was then transferred to a microcentrifuge tube and cells were pelleted at 4°C. Supernatants were stored at −20°C. Two additional extractions were then applied to the same membrane. Supernatants were combined before lyophilization and storage at −20°C.

### Liquid chromatography–tandem mass spectrometry

Compounds were quantified using an Agilent 1290 Infinity II UHPLC with an AB Sciex Qtrap 4000 at the Indiana University (IU) Mass Spectrometry Facility. Analytes were separated on a Waters BEH Amide column (2.1 × 150 mm, 2.5 μm particles) in HILIC mode. Dried samples were reconstituted in 53% mobile phase A plus 47% mobile phase B. Mobile phase A: 95% water, 5% acetonitrile, 20 mM NH_4_OH, 20 mM ammonium acetate with 5 μM medronic acid. Mobile phase B: 86% acetonitrile, 14% water, 20 mM NH_4_OH with 5 μM medronic acid. The gradient program (flow rate 0.3 ml/min) was: 100% B, 0 to 3 min; ramp 100% to 55% B, 3 and 8 min; hold at 55% B, 8 to 12 min; ramp to 100% B, 12 to 13 min; hold until 36 min. The program was applied for both positive and negative ion modes. QTrap 4000 was operated in multiple reaction monitoring mode using Analyst 1.7.1 software. Analytes were quantified using external calibration curves (Fig. S1). Unlabeled standards (purity ≥95%; all from Sigma-Aldrich, except for XMP from Santa Cruz Biotechnology and c-di-GMP from InvivoGen) were diluted in the same solvent as samples.

### Auxotroph bioassays

*R. palustris* monocultures (10 ml) were pelleted by centrifugation. Supernatants (3 ml) were syringe filtered (0.22 μm) into sterile, sealed, argon (Ar)-filled test tubes, and supplemented with 25 mM glucose, 10 mM NH_4_Cl, and 1% v/v cation solution before inoculation with washed *E. coli* Δ*purH* or *E. coli* Δ*pyrC* to ~0.003 OD_660_ and incubation overnight in the dark.

### Lysate preparation

Cell pellets from 10 ml monocultures were resuspended in 0.7 ml of MDC and transferred to 2-ml screw-cap tubes containing ~0.25 ml of 0.1 mm Zirconia/Silica beads (BioSpec Products). Cells were lysed at 4°C by eight rounds of bead-beating using a FastPrep-24 homogenizer (MP Biomedical) at max speed for 40 s per round. Lysates were centrifuged at 18 400 × *g* for 20 min at 4°C. Supernatants were then mixed with MDC and prepared as described for bioassays.

### Quantification of live and dead cells by flow cytometry

The live/dead BacLight Viability Kit (Invitrogen) was used per the manufacturer’s instructions with stationary phase monoculture cells resuspended in 25 mM 4-(2-hydroxyethyl)-1-piperazineethanesulfonic acid (HEPES) (pH 7.5) to ~10^6^ cells/ml. Samples were injected into a NovoCyte flow cytometer (Agilent) at 14 μl/min, excited at 488 nm, and emissions detected at 530/30 and 675/30 nm. Populations were analyzed using NovoExpress software (Agilent). Live cell populations were estimated using linear regression of live and dead cell mixtures. Dead cells were prepared by incubating with 70% v/v isopropanol for 1 h, then washed and resuspended in HEPES buffer.

### RNA purification

Monocultures were grown in MDC with 20 mM acetate and 10 mM NH_4_Cl to 0.4–0.8 OD_660_, chilled on ice, and then pelleted by centrifugation at 4°C. Supernatants were discarded and pellets were frozen using dry ice and stored at −80°C. RNA was extracted using the RNeasy Mini Kit (Qiagen) as per the manufacturer’s instructions, except the lysis step included bead beating as described above. RNA was quantified using a Nanodrop 1000 (Thermo Scientific) at the IU Physical Biochemistry Instrumentation Facility. RNA (20–25 μg) was treated with 4 U Turbo DNase (Ambion) in 100 μl at 37°C for 1 h. RNA was then cleaned using the RNeasy MinElute Cleanup Kit (QIAGEN), quantified, and adjusted with RNase-free water to 100–200 ng/μl.

### RNA sequencing

RNA (4 μg per sample) was processed by the IU Center for Genomics and Bioinformatics. rRNA was depleted using an Illumina Ribo-Zero Plus rRNA depletion kit. Libraries were prepared using an Illumina TruSeq Stranded mRNA HT kit. Sequencing was performed using an Illumina NextSeq 75-cycle, high-output run. A total of 27–31 million reads were obtained for each sample.

Analysis of differentially expressed genes was performed as described [25, 26] with minor modifications. Briefly, raw reads were preprocessed using Trim Galore v.0.6.6 (https://github.com/FelixKrueger/TrimGalore#readme), a Perl script employing Cutadapt v.1.18 [27], and FastQC v.0.11.5 [28] for trimming of adapter sequences, and removal of low-quality base calls, and quality control, respectively. Processed reads from both CGA0092 and TIE-1 were aligned to the *R. palustris* CGA009 reference genome (NCBI accession#: NC_005296) by HISAT2 v.2.1.0 with options −p, −dta, −no-spliced-alignment, and —rna-strandness RF [29]. Samtools v.1.15.1 was used to convert the sequence alignment and map (SAM) files output from HISAT2 into binary alignment and map (BAM) format. Aligned reads in BAM format were annotated and the transcript abundance was estimated using StringTie v.1.3.3b [25]. Transcript abundance tables were moved into R, and DESeq2 was used for differential gene expression analysis [30]. Genes unique to either strain could not be considered. Only genes with at least one sample containing ≥10 estimated transcript counts were included. Differentially expressed genes with an adjusted *P*-value <.05 and a |log2(fold-change)| > 2.0 were considered significant.

### Reverse transcription quantitative real-time PCR

cDNA was prepared from 2 μg RNA using random hexamer primers and SuperScript IV reverse transcriptase (RT; Invitrogen) following the manufacturer’s instructions. Standard curves were generated using gDNA. Each gDNA, cDNA, RT-minus, and no template control, and each sample was mixed with forward and reverse primers (300 nM each) and 1X SsoAdvanced Universal SYBR Green supermix (Bio-Rad) in a 96-well PCR plate (Eppendorf) for a total volume of 0.02 ml. The thermocycler program was: 98°C, 2 min; then 40 cycles of 98°C,15 s; 62°C, 40 s; 72°C, 30 s. The reaction was monitored using a Mastercycler ep *realplex* real-time PCR system (Eppendorf). Data were analyzed by *realplex* software using Noiseband. Primer efficiencies were 94%–100%. Specificities were validated by melting curves and by the presence of a single band on an agarose gel.

### Computational modeling

Diffusion of adenine was assessed by modifying a Monod model describing NH_4_^+^ cross-feeding cocultures [9, 31, 32]. The model omitted H_2_, CO_2_, and ethanol, which do not significantly impact cross-feeding [9, 31, 32], and N_2_ and NH_4_^+^ to reflect saturating NH_4_^+^. The adenine diffusion rate was modeled using Fick’s laws [33] and incorporated cell surface area [34], the permeability coefficient [35], and the difference between intracellular and extracellular adenine concentrations. The *E. coli* half-saturation constant for adenine was based on the average Km values for PurP and YicO transporters [36]. Equations and default parameters (Table S4) are in the supplementary materials. An R-studio version is at: github.com/McKinlab/Coculture-Mutualism/blob/master/adenineDiffusion_20231203.Rmd.

## Results

### Wild-type *R. palustris* CGA0092 supports *E. coli* purine auxotroph growth

Previously, we found that NH_4_^+^-excreting *R. palustris* Nx supported *E. coli* Δ*purK* purine auxotroph growth in coculture with N_2_ as the nitrogen source [12], suggesting that *R. palustris* Nx externalized purine(s) (Fig. 1A). However, Δ*purK* mutants were prone to suppressor mutations, which would complicate our experiments. Therefore, we made an *E. coli* Δ*purH* mutant, which should be less prone to suppression because PurH catalyzes two purine synthesis steps (Fig. 1B). We verified that *E. coli* Δ*purH* was an auxotroph (Fig. S2); adenine and adenosine, but not ATP, supported growth. *E. coli* Δ*purH* was also rescued in coculture with *R. palustris* Nx (Fig. 1C). We did not observe Δ*purH* suppressors during this study.

In previous cocultures, wild type (WT) *E. coli* depended on *R. palustris* Nx for NH_4_^+^ [9] (Fig. 1A, top). Adding NH_4_Cl decoupled the growth rates of the partners and led to coculture being overrun by *E. coli*, with growth trends resembling an *E. coli* monoculture [9] (Fig. 1C). We wondered if cocultures with *E. coli* Δ*purH* would respond similarly to NH_4_Cl (Fig. 1A, bottom). Instead of *E. coli* taking over, growth trends for NH_4_Cl-supplied *R. palustris* Nx + *E. coli* Δ*purH* cocultures resembled NH_4_^+^-cross-feeding cocultures, indicating that the *E. coli* growth rate was limited by adenine availability (Fig. 1C).

Purine externalization seemed costly, so we questioned whether it resulted from engineered mutations. We thus attempted to coculture *E. coli* Δ*purH* with WT *R. palustris* CGA0092. This strain also supported *E. coli* Δ*purH* in coculture (Fig. 1D). Thus, purine externalization is not a result of any engineered mutations.

### Purine externalization is not dependent on *E. coli* or growth conditions

In some cross-feeding systems, the recipient can influence metabolite externalization [37-39]. To test whether *E. coli* induces *R. palustris* purine externalization, we inoculated *E. coli* Δ*purH* into media supplemented with *R. palustris* monoculture supernatant. *E. coli* Δ*purH* grew proportionately to the amount of supernatant supplied (Fig. S3) and the *R. palustris* monoculture population size, regardless of the *R. palustris* growth phase (Fig. 1E). Thus, purine externalization occurs during exponential growth and is not induced by *E. coli*.

*R. palustris* can grow in diverse conditions that we hypothesized could affect purine externalization. We thus examined *E. coli* Δ*purH* growth with CGA0092 supernatants from photoautotrophic conditions and from conditions requiring aerobic or anaerobic respiration. *E. coli* Δ*purH* growth, a proxy for purine externalization, was roughly correlated with the amount of *R. palustris* growth rather than growth condition (Fig. 1F) or growth rate (Fig. S4).

### Purine cross-feeding supports coexistence

**Figure 2:**
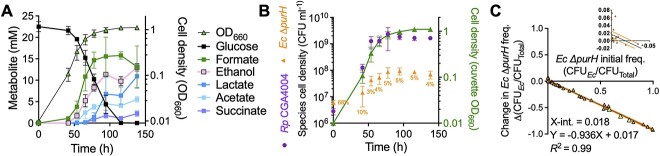
Purine cross-feeding supports coexistence; metabolic (A) and population trends (B) in batch *R. palustris* CGA4004 + *E. coli* Δ*purH* coculture with NH_4_Cl; percentages refer to the *E. coli* frequency; the initial frequency was 68 ± 10%; error bars = SD; *n*=3; some error bars are smaller than the symbols; (C) invasion-from-rare assay using the same strains and conditions to test the mutual invasion criterion for coexistence; an x-intercept between 0 and 1 is suggestive of coexistence; if the line falls entirely above or below the *x*-axis, then one population likely drives the other extinct; the cultures used in Fig. 2B are represented by yellow triangles; linear regression (central black line) ± 95% confidence intervals (outer orange lines) was applied; the inset graph is an enlarged portion of the same data to help visualize the x-intercept; initial *E. coli* Δ*purH* frequencies ranged from 0.00008 to 0.99898 *E. coli* CFU (CFU*_Ec_*)/ total CFU (CFU_Total_); change in frequency = (*E. coli* / (*E. coli* + *R. palustris*))_final_ – (*E. coli* / (*E. coli* + *R. palustris*))_initial_ [23].

We questioned how purine-cross-feeding cocultures compared to previous NH_4_^+^-cross-feeding cocultures. We therefore tracked fermentation products and populations in cocultures pairing CGA4004 + *E. coli* Δ*purH* with NH_4_Cl; CGA4004 gives similar coculture growth trends as CGA0092 (Fig. 1D) but has improved CFU accuracy due to its cell-aggregation deficiency. Cocultures accumulated organic acids that *R. palustris* can consume (consumable organic acids) (Fig. 2A), similar to NH_4_^+^-cross-feeding cocultures that used an *R. palustris* strain with a 3-fold high NH_4_^+^ excretion rate than the Nx strain, which caused *E. coli* to excrete organic acids faster than *R. palustris* could consume them [9]. *E. coli* Δ*purH* declined in frequency from 68 ± 10% (mean ± SD) to about ~3 ± 1% by the time growth stopped (Fig. 2B). This final frequency is similar to those observed with NH_4_^+^ cross-feeding [9, 31, 32, 37, 40].

Cocultures are most useful if they support coexistence. Coexistence is achieved when each population increases in frequency from rare (mutual invasion criterion) [21, 22]. We thus performed an invasion-from-rare assay, using a range of initial frequencies [23]. The assay is analogous to performing serial transfers, but explores more initial frequencies in parallel [23] and minimizes the influence from evolved subpopulations that can be enriched through serial transfers. Both species exhibited negative frequency-dependent selection (Fig. 2C); a decline in *E. coli* Δ*purH* frequency means that *R. palustris* increased in frequency. The x-intercept suggests that the strains coexist with an estimated *E. coli* Δ*purH* equilibrium of ~1.8 ± 0.9% (95% CI; Fig. 2C), assuming that the linear trend accurately represents the data at low *E. coli* frequencies [41]. Although the *E. coli* frequency is relatively low, the grown coculture includes tens-of-millions of *E. coli* cells/ml.

### *R. palustris* TIE-1 does not support *E. coli* Δ*purH* growth

**Figure 3:**
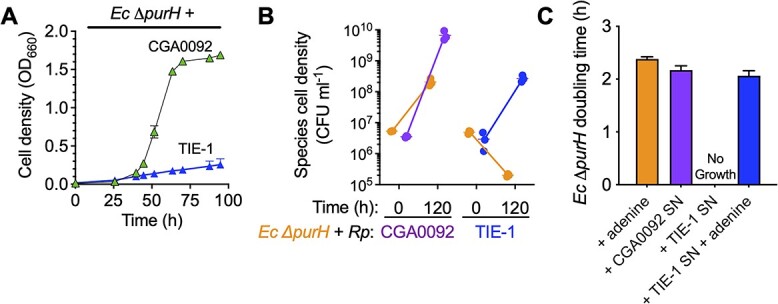
*R. palustris* (*Rp*) TIE-1 does not support *E. coli* (*Ec*) Δ*purH* auxotroph growth; (A) coculture growth curves of *E. coli* Δ*purH* with *R. palustris* CGA0092 or TIE-1; (B) comparison of initial and final population sizes by colony-forming units (CFUs) in cocultures pairing *E. coli* Δ*purH* with CGA0092 or TIE-1; (C) growth of *E. coli* Δ*purH* ± CGA0092 or TIE-1 supernatants ± 50 μM adenine; (A–C) error bars = SD; *n*=3; SN, supernatant.

*R. palustris* TIE-1 [42] is closely related to CGA0092; the two strains have identical 16S rRNA sequences and 5.28 Mb of CGA0092’s 5.46 Mb genome is shared at 97.9% identity [43]. We tested whether TIE-1 also externalizes purines by coculturing it with *E. coli* Δ*purH*. These cocultures exhibited linear growth (Fig. 3A). We previously observed linear growth in nitrogen-starved cocultures where non-growing *E. coli* fermented glucose for maintenance energy, resulting in a steady supply of organic acids for *R. palustris* [32]. Thus, TIE-1 likely grew on organic acids from non-growing *E. coli*. Indeed, *E. coli* Δ*purH* populations declined in coculture with TIE-1 (Fig. 3B). Results were not influenced by different CGA0092 versus TIE-1 growth traits; monoculture growth curves were similar (Fig. S5). The decline in *E. coli* Δ*purH* was not due to inhibitory factors produced by TIE-1; *E. coli* Δ*purH* grew in TIE-1 monoculture supernatants when supplemented with adenine (Fig. 3C). Thus, TIE-1 does not externalize enough purine(s) to support *E. coli* Δ*purH* growth.

### *R. palustris* CGA0092 externalizes adenine

**Figure 4:**
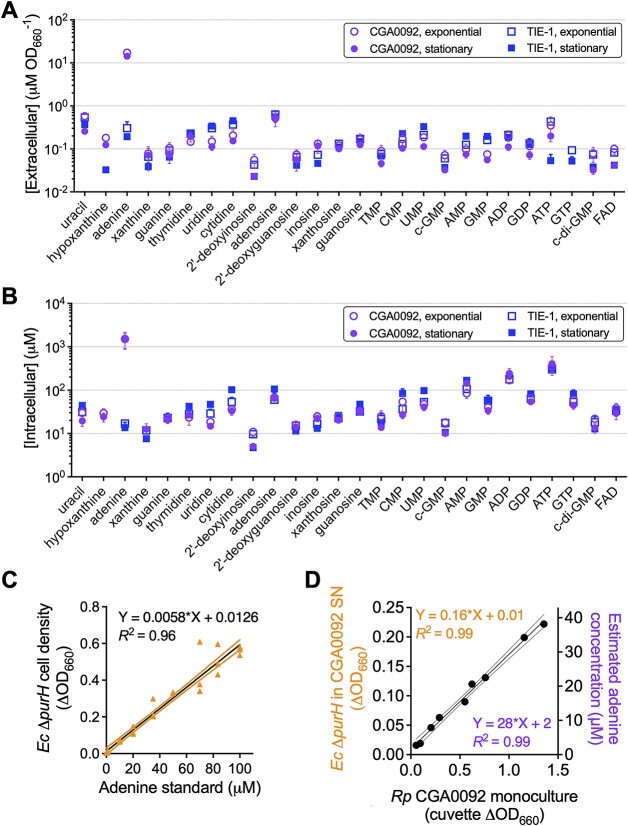
*R. palustris* CGA0092 externalizes adenine; extracellular (A) and intracellular (B) concentrations of nucleobase-containing compounds from monocultures of *R. palustris* CGA0092 and TIE-1; molecules are arranged by increasing molecular weight; error bars = SD; *n*=1–3 depending on whether molecules were detected in all samples; (C) standard curve for quantifying adenine in CGA0092 monoculture supernatants using an *E. coli* Δ*purH* bioassay; (D) estimated extracellular adenine (or bioavailable purines in general) in CGA0092 monoculture supernatants using the *E. coli* Δ*purH* bioassay; (C, D) outer lines for each linear regression analysis represent the 95% confidence interval; SN, supernatant.

We sought to identify the purine(s) externalized by CGA0092. Exploiting the lack of purine externalization by TIE-1, we used liquid chromatography–tandem mass spectrometry (LC–MS/MS) to compare nucleobase-containing compounds between the strains. For each strain, similar nucleobase concentrations were observed between exponential phase and stationary phase for both intracellular and extracellular samples, with the exception of adenine (Fig. 4A and B; Tables S5 and S6). Adenine was measured at 17 ± 2 μM/OD_660_ in CGA0092 exponential phase supernatants, which was 57-fold higher than in TIE-1 supernatants (0.3 ± 0.1 μM) (Fig. 4A). Intracellular adenine was 89-fold higher in CGA0092 (1516 ± 645 μM) compared to TIE-1 (17 ± 3 μM) (Fig. 4B).

We then developed a bioassay to facilitate external adenine quantification. By plotting *E. coli* Δ*purH* cell density versus adenine (Fig. 4C), we estimated 28 ± 2 μM adenine /CGA0092 OD_660_ (Fig. 4D), which was 1.6-fold higher than that determined by LC–MS/MS. Although the bioassay responds to other purines (Fig. S2), the discrepancy likely stems from differences in methodology. Aside from adenine, LC–MS/MS indicated that other purine levels were similar between CGA0092 and TIE-1 (Fig. 4A). Thus, if the other purines accounted for the discrepancy, we would expect *E. coli* Δ*purH* to grow in TIE-1 supernatants, but *E. coli* Δ*purH* growth would be 1.6-fold higher in CGA0092 supernatants. Instead, *E. coli* Δ*purH* does not grow in TIE-1 supernatants (Fig. 3C). Although it is possible that LC–MS/MS overlooked some purines, we conclude that *E. coli* Δ*purH* growth in CGA0092 supernatants is primarily due to adenine.

### Adenine externalization can be explained by diffusion across the membrane

**Figure 5:**
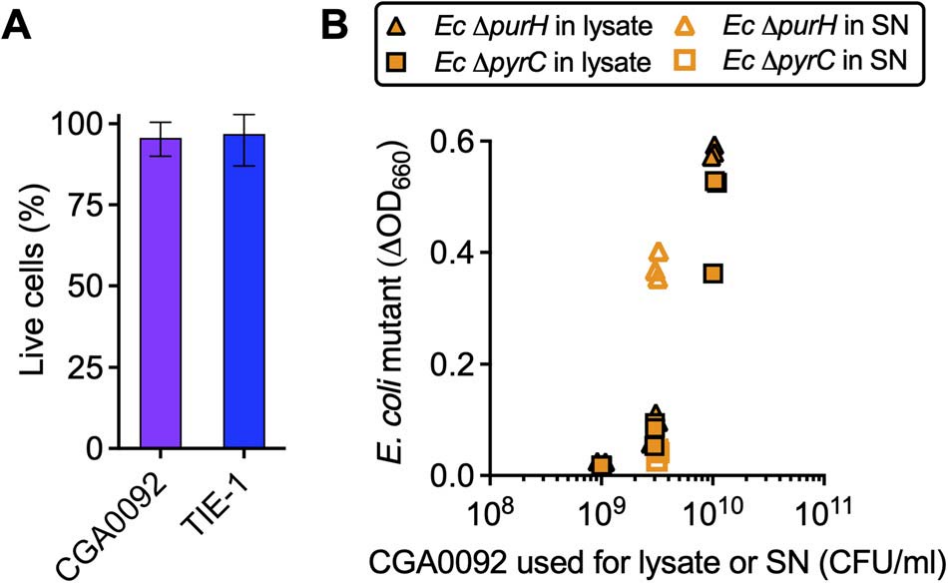
Cell lysis cannot explain *E. coli* Δ*purH* growth in CGA0092 supernatants; (A) live-dead stains of stationary phase CGA0092 and TIE-1 monocultures; approximately 3 × 10^7^ and 5 × 10^7^ cells were counted for CGA0092 and TIE-1, respectively; error bars, SD; (B) comparison of *E. coli* Δ*purH* (purine auxotroph) and Δ*pyrC* (pyrimidine auxotroph) growth in CGA0092 supernatants (open symbols) and lysates (closed symbols); each symbol represents a biological replicate (*n*=3); SN, supernatant.

We next pursued how adenine is externalized. We first addressed lysis by comparing live and dead cell frequencies in CGA0092 versus TIE-1 monocultures. Both strains had a similarly low frequency of dead cells, suggesting that lysis is not a major contributor to adenine externalization (Fig. 5A). A lack of adenine externalization via lysis was also supported by experiments where we grew *E. coli* Δ*purH* and a pyrimidine auxotroph control (Δ*pyrC*) with CGA0092 lysates. CGA0092 lysate only supported ~25% of the *E. coli* Δ*purH* growth observed in supernatants from an equivalent number of cells (Fig. 5B). Lysate also supported *E. coli* Δ*pyrC* growth. Thus, there is insufficient adenine in lysate to explain *E. coli* Δ*purH* growth in coculture (Fig. 5B).

**Figure 6:**
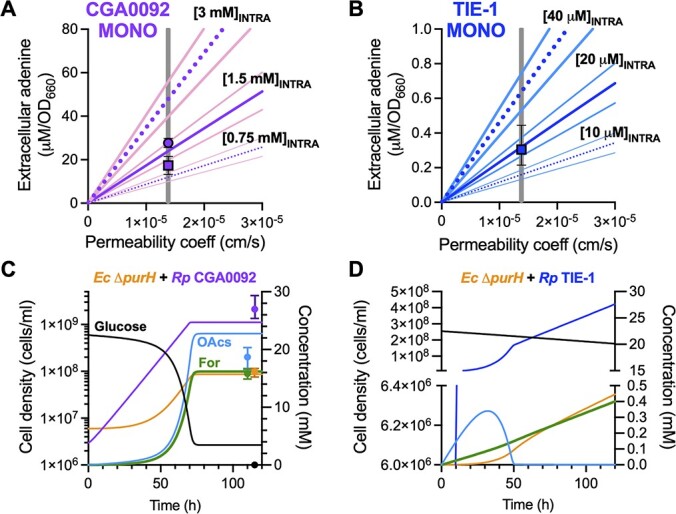
Simulations using adenine diffusion across a membrane accurately predict extracellular adenine levels; adenine externalization was simulated as a function of the adenine permeability coefficient, cell surface area, and the difference between intracellular and extracellular adenine concentrations using a Monod model (supplementary information); (A, B) simulations of monocultures where growth is limited by 10 mM organic acids were run for three different intracellular adenine concentrations (central purple [A] or dark blue [B] lines) and three different cell sizes (flanking pink [A] or light blue [B] lines represent the upper and lower bounds on cell size [34]; supplementary information) across a range of adenine permeability coefficients (coeff); vertical gray line, published adenine permeability coefficient [35]; square symbol, extracellular adenine measured by LC-MS/MS; round symbol, extracellular adenine measured by a bioassay; symbols were arbitrarily placed at the published adenine permeability coefficient; error bars = SD; *n*=3; (C, D) coculture simulations using the published adenine permeability coefficient, average cell size, intracellular adenine concentrations measured by LC-MS/MS (supplementary information); OAcs, organic acids; For, formate; (C) symbols represent final empirical values, arbitrarily placed to avoid overlap with the *y*-axis; error bars = range; *n*=3.

With lysis excluded, we addressed diffusion across the cytoplasmic membrane. We modified a model that simulated cocultures by replacing user-specified NH_4_^+^ excretion levels with a function describing adenine diffusion (see methods). The model accurately predicted extracellular adenine for both CGA0092 and TIE-1 monocultures using default parameters and for a range of realistic cell sizes [34], a 2-fold difference in intracellular adenine, and a > 2-fold change in permeability (Fig. 6A and B).

The model also accurately predicted the final *E. coli* population in coculture with CGA0092 (Fig. 6C). The model fell short of predicting observed *R. palustris* populations because it was overly sensitive to the inhibitory effect of pH, which limits the *R. palustris* population, glucose consumption, and organic acid consumption. However, when we took acid inhibition out of the model, the predicted *E. coli* population was still within the observed range (not shown). We did not assess quantitative predictions with TIE-1 cocultures because our model does not describe *E. coli* death, which occurs (Fig. 3B). However, the model still accurately predicted that TIE-1 adenine externalization cannot support substantial *E. coli* growth, resulting in linear TIE-1 population growth (Fig. 6D). Thus, adenine excretion can be explained by diffusion across a membrane, a literal definition of leakage [2]. For this reason, we did not address efflux proteins, though we cannot rule out their involvement.

### CGA0092 adenine externalization is likely influenced by low *apt* expression

**Figure 7:**
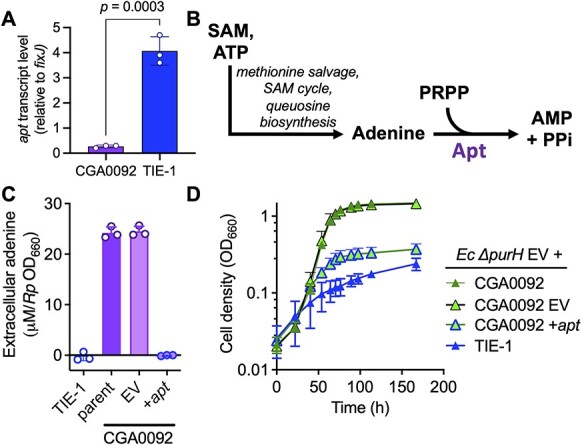
Ectopic expression of *apt* decreases CGA0092 adenine externalization; (A) transcript levels of *apt* in CGA0092 and TIE-1 relative to the house-keeping gene *fixJ* determined by RT-qPCR; (B) adenine salvage pathway, showing the location of the Apt enzyme; (C) extracellular purines (assumed to be adenine) in *R. palustris* supernatants with and without ectopic *apt* expression; adenine was measured using the *E. coli* Δ*purH* bioassay; (D) *R. palustris* + *E. coli* Δ*purH* coculture growth curves with and without ectopic *apt* expression; (A, C, D) error bars = SD; *n*=3; EV, empty vector pBBPgdh; +*apt*, constitutive expression vector pBBPgdh-*apt*.

To address why CGA0092 accumulates adenine, we again compared CGA0092 and TIE-1. Given their relatedness, we reasoned that RNAseq could reveal insightful gene expression differences. Indeed, of the 4757 genes compared, 64 had significantly higher transcript levels in TIE-1 and 517 had lower (Supplementary materials). Of interest was the low CGA0092 transcript levels for *apt* (TX73_RS22960, RPA4492), encoding the purine salvage enzyme adenine phosphoribosyltransferase (Apt). RT-qPCR verified the difference (Fig. 7A).

We hypothesized that low CGA0092 *apt* expression creates a bottleneck, leading to adenine accumulation and leakage (Fig. 7B). If so, higher expression should alleviate the bottleneck. Thus, we expressed CGA0092 *apt* under a constitutive promoter from a plasmid and measured extracellular adenine using the bioassay. Supernatants from CGA0092 monocultures with and without an empty vector had similar adenine levels, whereas adenine was undetectable when CGA0092 expressed *apt* from a plasmid (Fig. 7C). Similar trends were seen in cocultures; the CGA0092 empty vector control gave growth curves similar to coculture with CGA0092, whereas CGA0092 expressing *apt* from a plasmid resulted in poor coculture growth (Fig. 7D).

### Orientation of *apt* does not always indicate purine externalization

**Figure 8:**
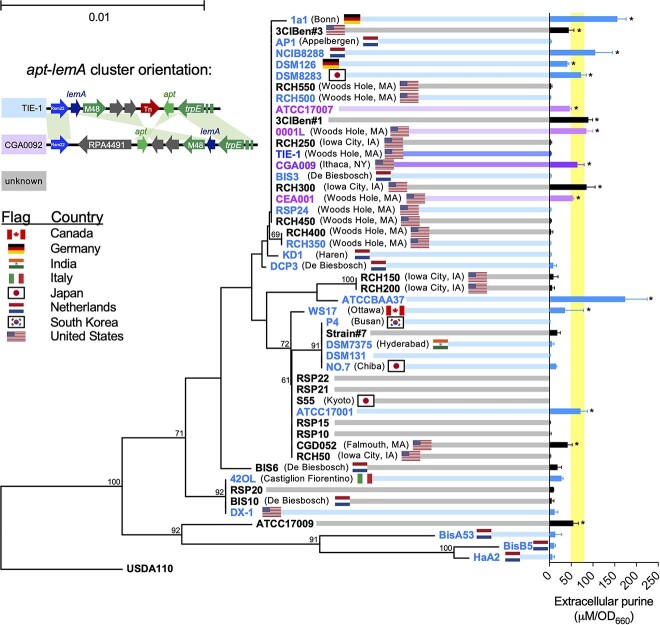
Diverse *R. palustris* strains externalize purines, regardless of *apt* orientation; phylogenetic relationships of 49 *R. palustris* strains tested for adenine excretion based on 16S rRNA sequences; bracketed text and flags indicate isolation location where known; bootstrap values (100 replicates) are at branch points (only showing values > 50); the bar represents 0.01 substitutions per site; *Bradyrhizobium diazoefficiens* USDA110 was used to root the tree; purple or blue tree shading indicates CGA0092 or TIE-1 *apt* gene orientation, respectively; gray shading indicates unknown *apt* gene orientation; right; purines were measured in *R. palustris* monoculture supernatants using the *E. coli* Δ*purH* bioassay; yellow shading indicates the CGA0092 standard deviation; ^*^ significantly more purine than TIE-1 from one-way ANOVA with a Dunnett correction for multiple comparisons; *P* < .1; error bars = SD, *n* = 3; these data are also organized by *apt* gene cluster orientation in Fig. S8; inset; CGA0092 and TIE-1 *apt-lemA* clusters shown to scale; other bacteria with the TIE-1 orientation do not have the transposon (Tn; Fig. S7).

We questioned why CGA0092 *apt* expression is low. TIE-1 *apt* and the 73-nucleotide upstream intergenic region match that in CGA0092. However, *apt* is part of a gene cluster that is oppositely oriented in CGA0092 versus TIE-1 (Fig. 8 inset). We hypothesized that gene orientation affected *apt* expression. This hypothesis was supported by RNAseq data showing that *lemA,* on the other side of the cluster, had 2.2-fold higher transcript levels in CGA0092 (Fig. S6 and Supplementary materials), suggesting read-through from *trpE*.

To explore whether *apt* orientation can explain adenine externalization, we used the bioassay to measure extracellular adenine (and possibly other purines) from 49 *R. palustris* strains (Fig. 8 and Fig. S8). Sixteen strains externalized significantly more purine than TIE-1, suggesting that *R. palustris* purine externalization is relatively common. The CGA0092-like *apt* orientation was always associated with purine externalization. However, some strains with a TIE-1-like orientation also externalized purine. Thus, additional factors beyond *apt* orientation can contribute to purine externalization.

## Discussion

We determined that *R. palustris* CGA0092 externalizes enough adenine to support an *E. coli* purine auxotroph. Based on RNAseq, mutational approaches, and computational modeling, we propose that adenine accumulates due to a bottleneck in adenine salvaging and then leaves the cell by diffusion across the cytoplasmic membrane. Purine externalization was also observed in 15 other *R. palustris* isolates, suggesting that this phenomenon could occur in nature (Fig. 8 and Fig. S8). *R. palustris* purine externalization is another example of externalized valuable metabolites, joining prior examples like amino acids [44-46] and vitamins [38, 46-48].

### What is the role of *R. palustris* adenine externalization?

*R. palustris* strains from diverse regions exhibited purine externalization, suggesting a possible physiological, signaling, or ecological role. It is also possible that the origins and maintenance of purine externalization involve interplay between physiological and ecological roles.

Adenine externalization could be analogous to uracil externalization*,* which helps *E. coli* maintain growth rates in response to perturbations to pyrimidine pools [49]. For CGA0092*,* adenine externalization is a feature of exponential growth across diverse conditions (Fig. 1E and F). Thus, if adenine externalization maintains homeostatic metabolite levels, it is constitutive and potentially effective at maintaining other nucleobase-containing metabolite levels, which were similar to those in TIE-1 (Fig. 4B).

Adenine externalization could also result from overflow metabolism, triggered by more carbon than normally experienced in nature [50, 51]. If true, one might expect a correlation between growth rate and adenine externalization [52]. However, we did not observe a strong correlation, at least when growth rate was determined by the growth condition (Fig. S4). The observation that some closely related strains do not externalize adenine (Fig. 8), despite having similar growth trends (Fig. S5), also argues against overflow metabolism due to carbon excess.

Extracellular adenine could also participate in signaling and/or cross-feeding. Some pathogens externalize ATP as a signal molecule for its immunosuppressing effects [53]. *R. palustris* has features that suggest inter-organismal relationships. Similar to its reliance on other organisms to convert sugars into organic acids, *R. palustris* relies on lignolytic organisms for consumable lignin monomers [54]. Lignin also plays into *R. palustris* quorum sensing (e.g. density-dependent intercellular signaling). CGA009 can only make its quorum sensing signal when supplied with lignin monomers, hinting at a relationship with plants or lignolytic fungi [55]. Perhaps purine externalization by *R. palustris* isolates likewise hints at an inter-organismal relationship (Fig. 8).

### Mechanisms of metabolite externalization

Although it is often assumed that metabolites passively leak from bacteria, transporters are likely involved for most charged and polar molecules [2]. Some purine efflux proteins are known [56, 57]. However, our model suggests that adenine, an uncharged molecule and the most hydrophobic of those we measured by LC–MS/MS, can diffuse across membranes and, if the intracellular adenine concentration is large enough, that leakage can lead to cross-feeding. If a nearby microbe can reciprocate, and close proximity is maintained, then cross-feeding could become subject to selection [3, 4, 6].

Although diffusion can explain adenine externalization, we cannot rule out efflux proteins. Involvement of efflux proteins might be suggested by the similar adenine concentrations observed between exponential and stationary phase; adenine externalization halted with growth (Fig. 4). However, the similar levels could also be explained if leakage and uptake reached a steady state in stationary phase, or if stationary phase membrane composition, which includes cyclopropane fatty acids [58], limited leakage.

### Elusive signatures for metabolite externalization

Predictions of cross-feeding interactions from genomic data are often based on the absence of biosynthetic genes [5, 59]. Such missing genes suggest that an organism competitively or cooperatively acquires metabolites from a neighbor. However, when considering externalization of communally valuable metabolites, we lack even suggestive genetic signatures.

We hypothesized that *apt* orientation was a signature for adenine externalization. Whereas the CGA0092 orientation was always correlated with purine externalization, all strains with this orientation were also closely related (Fig. 8). Some strains that had the opposite *apt* orientation also externalized adenine, or possibly other purine(s). Thus, *apt* orientation might be one driver of purine externalization but other unknown factors can also be sufficient. Even if *apt* orientation is a signature for purine externalization, the synteny of the reversible gene cluster is not conserved outside of *R. palustris*.

Our study highlights a challenge in predicting metabolite-externalizing microbes. Genomic signatures could be more diverse than the repertoire of cross-fed metabolites. Metabolite externalization can also be conditional [2, 7, 8, 38, 39, 49, 60] and need not even require a genetic signature. Previously, we found that enhanced metabolite acquisition by a recipient was sufficient to establish cross-feeding; the genetic signature was in the recipient, not the producer [37]. With such confounding elements, mechanistic studies into microbial interactions will remain essential to understand the factors governing metabolite externalization before we can make accurate predictions about cross-feeding from the wealth of available genomic information [7, 8, 60].

## Supplementary Material

SI_Purine_cross-feeding_manuscript_2024_02_rev_wrae034

Purine_crossfeeding_SUPP_DESeq_wrae034

## Data Availability

RNAseq datasets are in the NCBI Sequence Read Archive under BioProject accession number PRJNA1029518 (www.ncbi.nlm.nih.gov/bioproject). All other data for this study are in this article and its supplementary information files.
