# Supplementary material for: Bacterial adenine cross-feeding stems from a purine salvage bottleneck
Source: ISME J. 2024 Mar 7;18(1):wrae034. doi: 10.1093/ismejo/wrae034 (PMC10976475; doi:10.1093/ismejo/wrae034)
Supplement: SI_Purine_cross-feeding_manuscript_2024_02_rev_wrae034 [file si_purine_cross-feeding_manuscript_2024_02_rev_wrae034.pdf]

SUPPLEMENTARY MATERIAL FOR

Bacterial adenine cross-feeding stems from a purine salvage bottleneck

Ying-Chih Chuang[1,2], Nicholas W. Haas[1], Robert Pepin[3], Megan Behringer[4], Yasuhiro Oda[5], Breah LaSarre[1a], Caroline S. Harwood[5], and James B. McKinlay[1*]

[1]Department of Biology, Indiana University, Bloomington, IN
[2]Biochemistry Program, Indiana University, Bloomington, IN
[3]Department of Chemistry, Indiana University, Bloomington, IN
[4]Department of Biological Sciences, Vanderbilt University, Nashville, TN
[5]Department of Microbiology, University of Washington, Seattle, WA

*Corresponding author: 1001 E 3rd Street, Bloomington, IN 47405, USA;
Email: jmckinla@iu.edu

Current address:
[a] Department of Plant Pathology, Entomology, and Microbiology, Iowa State University, Ames, Iowa, USA

Contents:

Cross-feeding model for testing the validity of adenine diffusion
Figures S1-S8
Tables S1-S6
References

CROSS-FEEDING MODEL FOR TESTING THE VALIDITY OF ADENINE DIFFUSION

(1) *E. coli* growth rate (h^{-1}):

$$\mu_{Ec} = \mu_{EcMAX} \cdot [G/(k_G + G)] \cdot [P/(k_P + P)] \cdot [b_{Ec}/(b_{Ec} + 10^{(f+C)})]$$

(2) *R. palustris* growth rate (h^{-1}):

$$\mu_{Rp} = \mu_{RpMAX} \cdot [C/(k_C + C)] \cdot [b_{Rp}/(b_{Rp} + 10^{(f+C)})]$$

(3) change in glucose concentration (mM/h):

$$dG/dt = -\mu_{Ec} \cdot Ec/Y_G - Ec \cdot [G/(k_G + G)] \cdot [10/(10 + 1.09^{(1000 \cdot \mu Ec}))] \cdot (b_{Ec}/(b_{Ec} + 10^{(f+C)})) \cdot ((100/(100 + 6^C)) \cdot (r_C + r_f))$$

(4) change in purine (adenine) concentration (mM/h):

$$dP/dt = -\mu_{Ec} \cdot Ec/Y_P + Rp \cdot (IP\text{-}P) \cdot Perm \cdot SA \cdot [C/(k_C + C)] \cdot [b_{Rp}/(b_{Rp} + 10^{(f+C)})]$$

(5) change in organic acid concentration (mM/h):

$$dC/dt = -\mu_{Rp} \cdot Rp/Y_C + \mu_{Ec} \cdot Ec \cdot Fc + Ec \cdot [G/(k_G + G)] \cdot [10/(10 + 1.09^{1000 \cdot \mu Ec})] \cdot (b_{Ec}/(b_{Ec} + 10^{(f+C)})) \cdot r_C \cdot 100/(100 + 6^C)$$

(6) change in formate concentration (mM/h):

$$df/dt = \mu_{Ec} \cdot Ec \cdot Ff + Ec \cdot [G/(k_G + G)] \cdot [10/(10 + 1.09^{1000 \cdot \mu Ec})] \cdot (b_{Ec}/(b_{Ec} + 10^{(f+C)})) \cdot r_f \cdot 100/(100 + 6^C)$$

(7) change in *R. palustris* population (cells/ml/h):

$$dRp/dt = Rp \cdot \mu_{Rp}$$

(8) change in *E. coli* population (cells/ml/h):

$$dEc/dt = Ec \cdot \mu_{Ec}$$

where,

μ is the specific growth rate of the indicated species (h^{-1}).
μ_{MAX} is the maximum specific growth rate of the indicated species (h^{-1})
G is the extracellular glucose concentration (mM)
P is the extracellular purine (adenine) concentration (mM)
IP is the intracellular purine (adenine) concentration (mM)

C is the extracellular organic acid concentration (mM)
f is the extracellular formate concentration (mM)
k is the half saturation constant (km) for the indicated substrate (mM).
Ec is the *E. coli* cell density (cells/ml)
Rp is the *R. palustris* cell density (cells/ml)
b modulates sensitivity to inhibitory effects of accumulated organic acids (mM).
Y is the growth yield on the indicated substrate (cells / µmol).
F is the excretion value for the indicated compound during growth (µmol / cell)
r is the growth-independent excretion rate (µmol / cell / h)
Perm is the permeability coefficient for adenine (cm / s) (1)
SA is the cell surface area (cm^2)

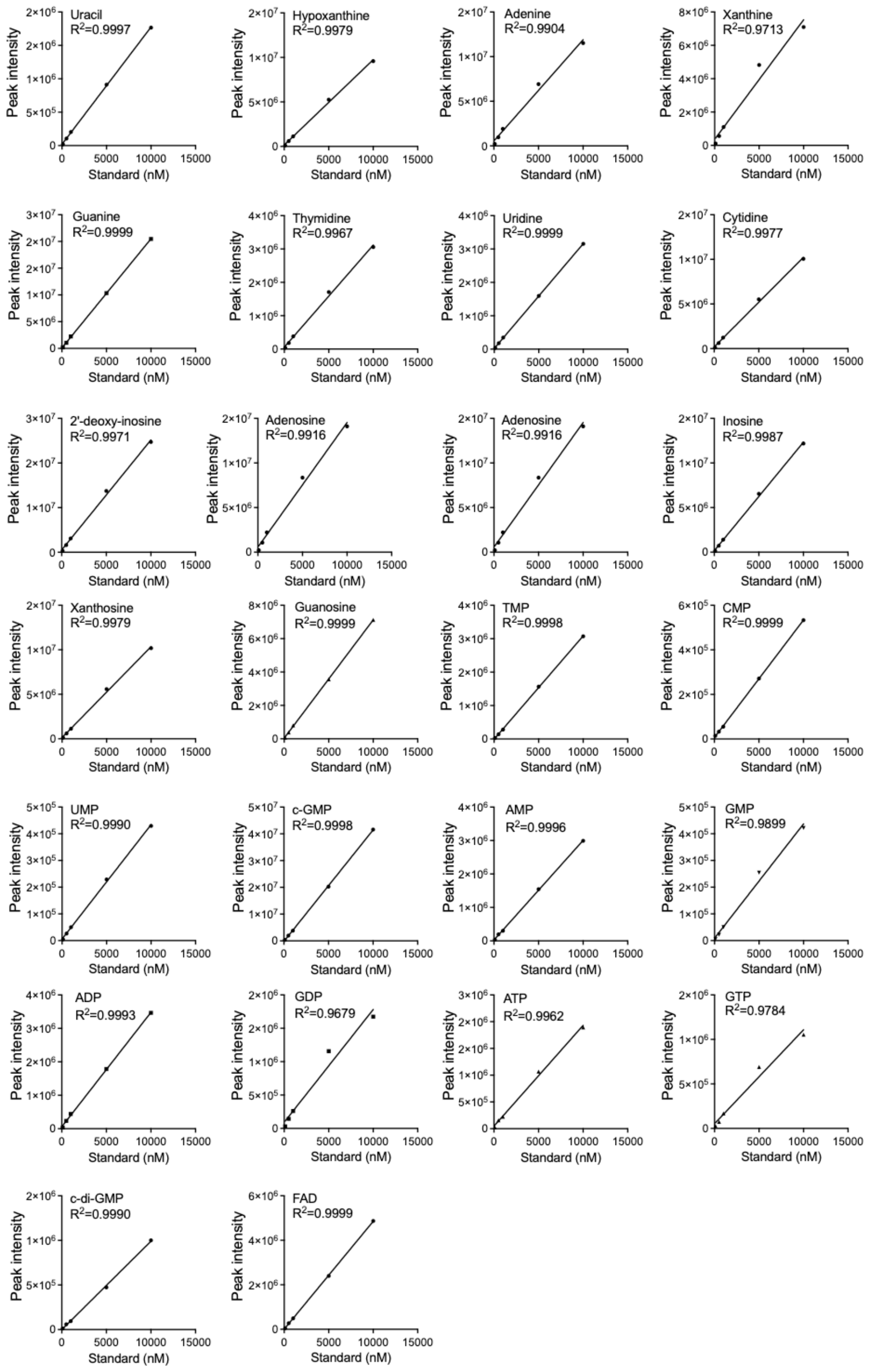

Fig S1. Standard curves used in LC-MS-MS analyses.

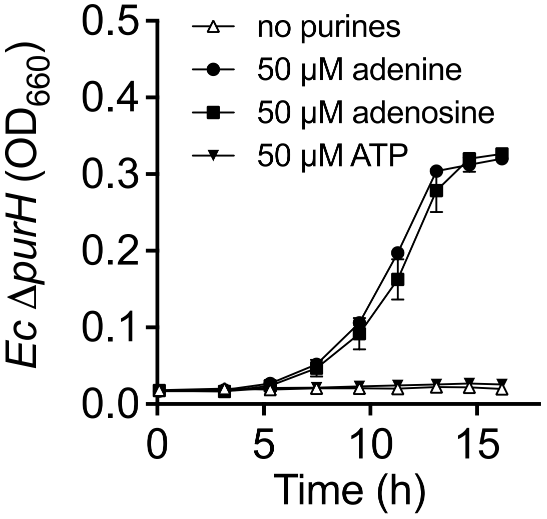

Fig. S2. ***E. coli*** **(*Ec*) Δ*purH*, requires purines for growth in monoculture.** Error bars = SD; n = 3. Some error bars are smaller than the symbols.

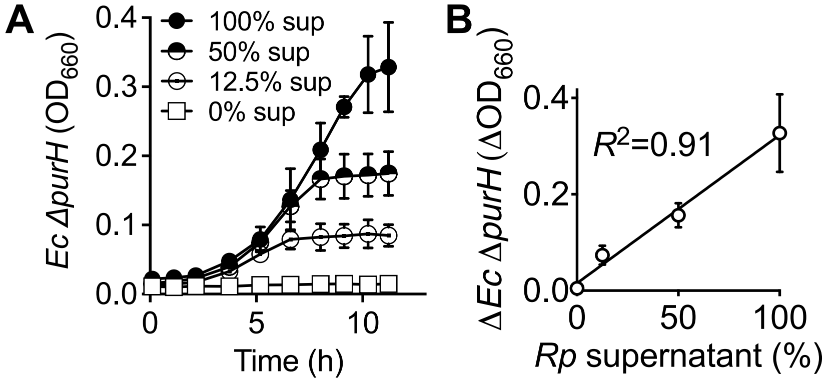

Fig. S3. ***R. palustris*** **(*Rp*) CGA4005 supernatants support** ***E. coli*** **(*Ec*) Δ*purH*** **monoculture growth.** Error bars = SD, n = 3. Some error bars are smaller than the symbols. **A.** *E. coli* Δ*purH* monoculture growth curves in media supplemented with difference amounts of *R. palustris* CGA4005 monoculture supernatant **B.** Linear regression of *E. coli* Δ*purH* monoculture growth and the amount of *R. palustris* CGA4005 supernatant provided.

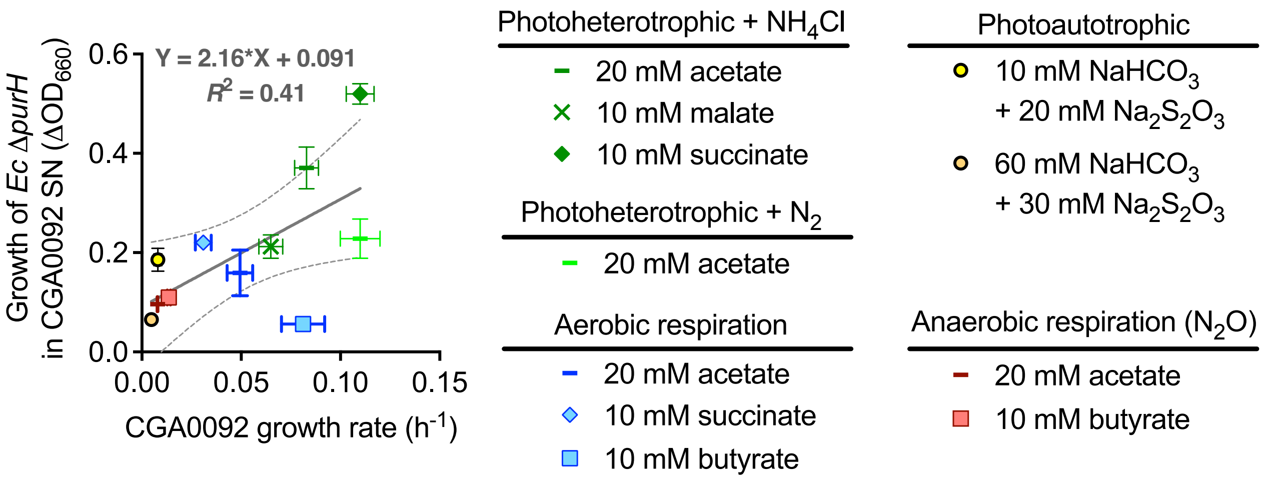

Fig S4. Purine externalization does not correlate well with CGA0092 growth rate across diverse growth conditions. Growth of *E. coli* (*Ec*) Δ*purH* in supernatant samples taken from stationary-phase CGA0092 monocultures grown under various growth conditions. Each data point represents the mean of three to six biological replicates +/- SD. Linear regression (gray solid line) +/- 95% confidence intervals (dashed lines) was applied to all samples across all conditions. SN, supernatant.

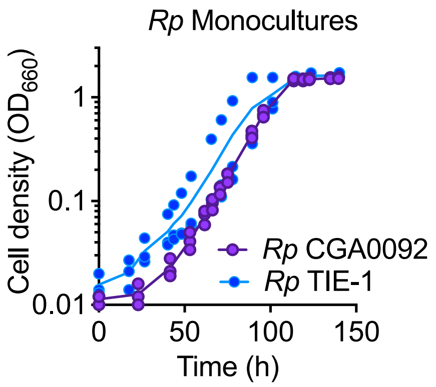

Fig. S5. Monoculture growth trends are similar for *R. palustris (Rp)* CGA0092 and TIE-1. Data points from all three biological replicates are shown.

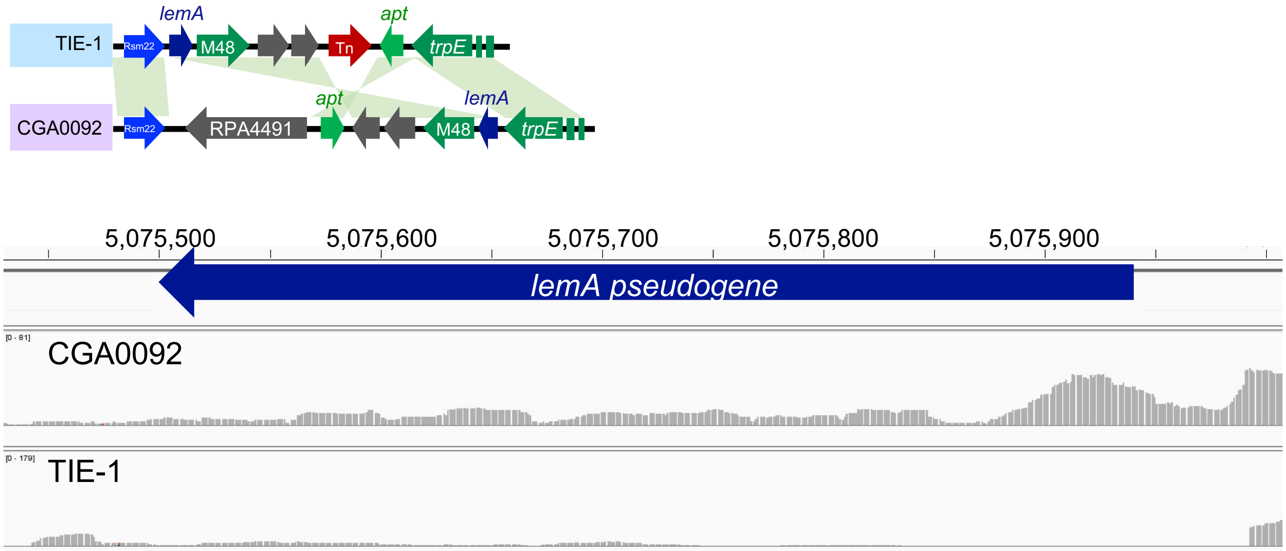

Fig. S6. CGA0092 exhibits higher *lemA* expression than TIE-1. Top, orientation of the *lemA-apt* cluster in CGA0092 and TIE-1. Bottom, sequencing reads (height of gray bars) across the *lemA* gene in each strain. See the supplementary RNAseq data for the corresponding differential expression values that estimate a 2.2-fold higher transcript levels in CGA0092.

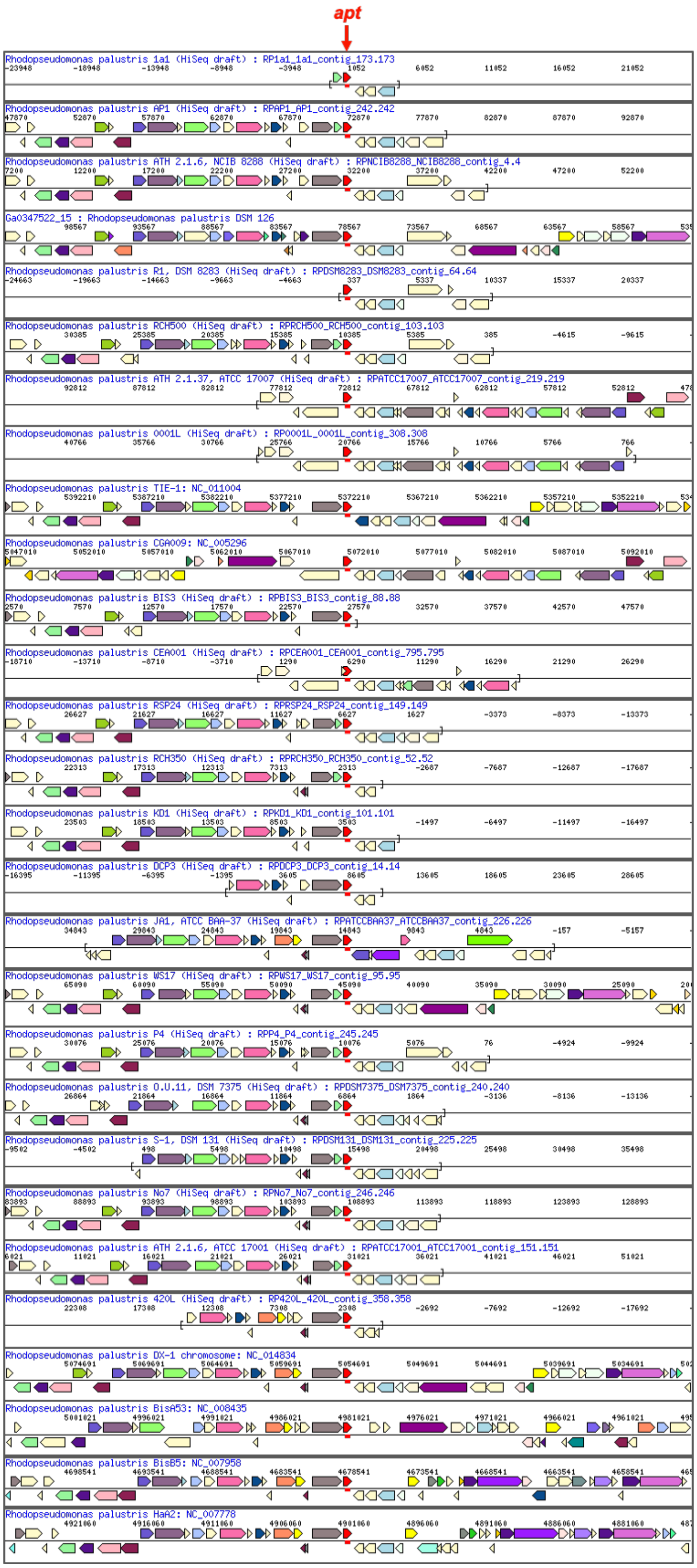

Fig. S7. Gene neighborhoods for *apt* (red). Images are from the Joint Genome Institute's Integrative Microbial Genomes & Microbiomes Gene Neighborhoods tool.

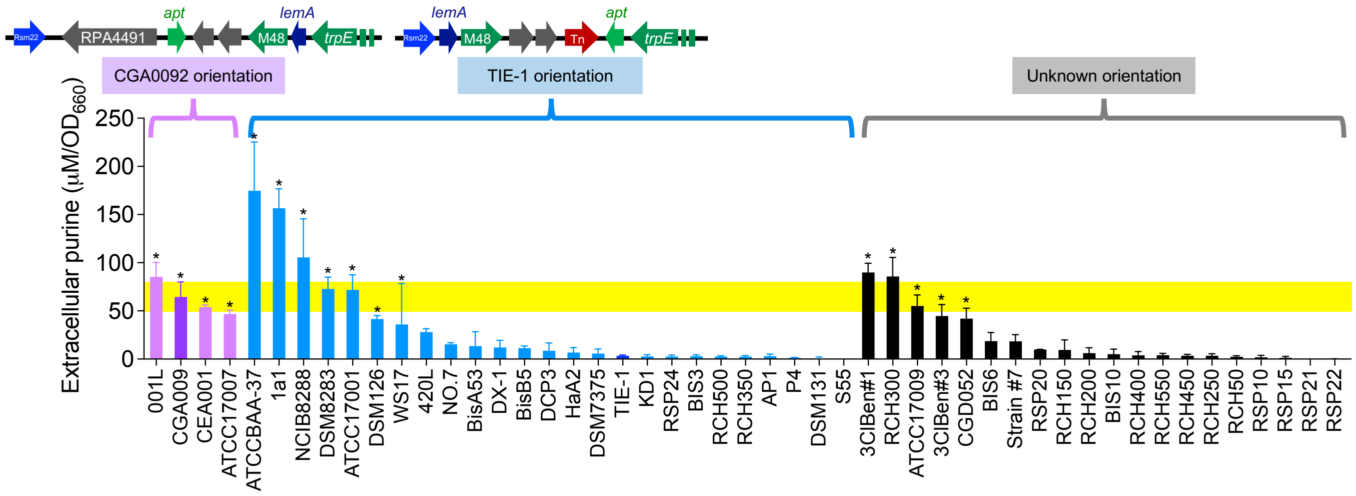

Fig S8. Purine externalizing by *R. palustris* strains organized by *apt* orientation. Purple and blue shading indicates CGA0092 or TIE-1 *apt* gene orientation, respectively. Gray shading indicates unknown *apt* gene orientation. Purines were measured in *R. palustris* monoculture supernatants using the *E. coli* Δ*purH* bioassay. Yellow shading indicates the CGA0092 standard deviation. *, significantly more purine than TIE-1 from One-way ANOVA with a Dunnett correction for multiple comparisons; $p < 0.1$. Error bars = SD, n = 3. **Top.** CGA0092 and TIE-1 *apt-lemA* clusters shown to scale. Other bacteria with the TIE-1 orientation do not have the transposon (Tn; Fig S7).

Table S1. Strains used in this study.

Strain	Genotype; 16S rRNA GenBank accession number	Source or Reference
R. palustris		
CGA0092*	Type strain; derivative of CGA001 with spontaneous chloramphenicol resistance	(2)
CGA4004	CGA0092 Δ*hupS* Δ*rpa2750*	(3)
CGA4005	CGA4004 NifA*; a strain with constitutive nitrogenase activity; named Nx in the previous study	(3)
TIE-1*	Environmental isolate	(4)
DX-1*	Environmental isolate	(5)
1a1*	Environmental isolate	(6)
3ClBen#3	Environmental isolate; OR656700	Harwood collection
AP1*	Environmental isolate	(6)
NCIB8288*	Type strain	DSMZ, (6)
DSM126*	Type strain	DSMZ, (6)
DSM8283*	Type strain	DSMZ, (6)
RCH550	Environmental isolate; OR656702	Harwood collection
RCH500*	Environmental isolate	(6)
ATCC17007*	Type strain	ATCC, (6)
3ClBen#1	Environmental isolate; OR656699	Harwood collection
0001L*	Environmental isolate	(6)
RCH250	Environmental isolate; OR656697	Harwood collection
BIS3*	Environmental isolate	(6)
RCH300	Environmental isolate; OR656698	Harwood collection
CEA001*	Environmental isolate	(6)
RSP24*	Environmental isolate	(6)
RCH450	Environmental isolate; OR656701	Harwood collection
RCH400	Environmental isolate; OR659638	Harwood collection
RCH350*	Environmental isolate	(6)
KD1*	Environmental isolate	(6)
DCP3*	Environmental isolate	(6)
RCH150	Environmental isolate; OR656709	Harwood collection
RCH200	Environmental isolate; OR656708	Harwood collection
ATCCBAA37*	Type strain	ATCC
WS17*	Environmental isolate	(7)
P4*	Environmental isolate	(8)
Strain#7	Environmental isolate; OR656710	Harwood collection
DSM7375*	Type strain	DSMZ
DSM131*	Type strain	DSMZ
No.7*	Environmental isolate	(9)
RSP22	Environmental isolate; OR656707	Harwood collection

RSP21	Environmental isolate; OR656706	Harwood collection
S55*	Environmental isolate	(9)
ATCC17001*	Type strain	ATCC
RSP15	Environmental isolate; OR656705	Harwood collection
RSP10	Environmental isolate; OR656704	Harwood collection
CGD052	Environmental isolate; OR656703	Harwood collection
RCH50	Environmental isolate; OR656696	Harwood collection
BIS6	Environmental isolate; OR656711	(10)
42OL*	Environmental isolate	(11)
RSP20	Environmental isolate; OR656713	Harwood collection
BIS10	Environmental isolate; OR656712	(10)
ATCC17009	Type strain; OR656714	ATCC
BisA53*	Environmental isolate	(12)
BisB5*	Environmental isolate	(12)
HaA2*	Environmental isolate	(12)
E. coli		
MG1655	Wild-type K-12	(13)
MG1655 Δ*purH*::Km^R	Δ*purH*::Km^R; mutant allele came from Keio strain JW3970; Referred to as *E. coli* Δ*purH* in the paper.	this study
MG1655 Δ*pyrC*::Km^R	Δ*pyrC*::Km^R; mutant allele came from Keio strain JW1049; Referred to as *E. coli* Δ*pyrC* in the paper.	this study
NEB10β	DH10B derivative; *Δ(ara-leu) 7697 araD139 fhuA ΔlacX74 galK16 galE15 e14- ϕ80dlacZΔM15 recA1 relA1 endA1 nupG rpsL* (Str^R) *rph spoT1 Δ(mrr-hsdRMS-mcrBC)*	New England Biolabs
JW3970	Δ*purH*:: Km^R; Keio knockout strain	(14)
JW1049	Δ*pyrC*:: Km^R; Keio knockout strain	(14)

* 16S rRNA gene sequence is obtained from the Integrated Microbial Genome (IMG) database (https://img.jgi.doe.gov/).

Table S2. Plasmids used in this study.

Plasmids	Genotype or primer sequence (5’ to 3’), description	Source or Reference
pKD46	Red recombinase expression plasmid; to replace target genes in *E. coli* with PCR products from the Keio collection	(15)
pCP20	Yeast Flp recombinase expression plasmid; to remove the Km resistance cassette in *E. coli*	(16)
pBBPgdh	Gm^R; pBBR1MCS-5 with a constitutive RPA0944 promoter	(17)
pBBPgdh-apt	Gm^R; pBBPgdh containing CGA009 *apt* (RPA4492) and the native ribosomal biding site downstream of RPA0944 promoter	This study

Table S3. Primers used in this study.

Primer	Sequence (5’-3’)	Description
YCC27	CCC TAT TTG AAC CAG GCA TTA CGC	5’ of Δ*pyrC*::*Km* in JW1049
YCC28	CGC TGT TTA TCT TCT TTT GTC GCG CC	3’ of Δ*pyrC*::*Km* in JW1049
YCC29	GCG CAA ACG TTT TCG TTA CAA TGC G	5’ of Δ*purH*::*Km* in JW3970
YCC30	TGC ATT ACC CGG AGC AAC	3’ of Δ*purH*::*Km* in JW3970
YCC72	GAG GTC GAC GGT ATC GAT AAG CTC CGT AAC GAA AGA CCC GCG CCG	5’ of RPA4492 (*apt),* overlapping with pBBPgdh
YCC73	AAT TGG AGC TCC ACC GCG GTG GCG GAG AGT TAG GCA GGA GCG AGC	3’ of RPA4492 (*apt),* overlapping with pBBPgdh
YCC74	ATC GGC GCG GGT CTT TCG TTA CGG AGC TTA TCG ATA CCG TCG ACC	Amplify pBBPgdh overlapping with 5’ of RPA4492 (*apt)*
YCC75	ATT GAG CTC GCT CCT GCC TAA CTC TCC GCC ACC GCG GTG GAG CTC	Amplify pBBPgdh overlapping with 3’ of RPA4492 (*apt)*
YCC76	ATC ATC TTC CGC GAC ATC AC	Forward qPCR primer for *apt*
YCC77	CCT TGT CGA TCT TCG AAC CC	Reverse qPCR primer for *apt*
YCC78	TCG CCG AGG TCG AAG TA	Forward qPCR primer for lemA
YCC79	GTA CAA CAC CGG CAT CCA	Reverse qPCR primer for *lemA*
YCC80	TAA GGG AAC CGT GCA TGT G	Forward qPCR primer for *fixJ*
YCC81	GGA TTC GTA CAG CTT GAC CTC	Reverse qPCR primer for *fixJ*

Table S4. Default parameters used to simulate on adenine cross-feeding cocultures.

Parameter	Value	Description (Units); Source
μ_{EcMAX}	0.2800	*E. coli* max growth rate (h^{-1}); (18)
μ_{RpMAX}	0.0875	*R. palustris* max growth rate (h^{-1}); Monoculture with acetate and NH_4Cl
G	25	extracellular glucose (mM)
P	1×10^{-7}	extracellular adenine (mM); non-zero to initiate growth
IP	1.5	CGA0092 intracellular adenine (mM); LC-MS-MS measurements; use 0.02 mM for TIE-1
C	1×10^{-7}	extracellular consumable organic acids (mM); non-zero to initiate growth; set to 10 mM for *R. palustris* monocultures
f	0	extracellular formate (mM)
k_G	0.02	*E. coli* half-saturation constant (Km) for glucose (mM); (19)
k_P	0.004	*E. coli* half-saturation constant (Km) for adenine (mM); averaged for PurP and YicO (20)
k_C	0.01	*R. palustris* half-saturation constant (Km) for consumable organic acids (mM); Assumed
Perm	1.38×10^{-5}	Adenine permeability coefficient (cm / s); (1)
SA	5.1×10^{-8}	*R. palustris* surface area for a 3.25 µm long cell (cm^2); (21)<br>Upper bound: 4.24×10^{-8} cm^2 for a 2.70 µm long cell<br>Lower bound: 5.97×10^{-8} cm^2 for a 3.80 µm long cell
Ec	6×10^6	Initial *E. coli* cell density (cells / ml); Fig 2 time-course
Rp	3×10^6	Initial *R. palustris* cell density (cells / ml); Fig 2 time-course
b_{Ec}	10^{43}	resistance of *E. coli* to acid, reflects medium buffering (mM); (18)
b_{Rp}	10^{32}	resistance of *R. palustris* to acid, reflects medium buffering (mM); (18)
Y_G	4.4×10^6	glucose-limited *E. coli* growth yield (cells / µmol); Fig 2 time-course
Y_P	6×10^9	adenine-limited *E. coli* growth yield (cells / µmol); Fig 4 bioassay standard curve
Y_C	2×10^8	organic acid-limited *R. palustris* growth yield (cells / µmol); acetate-limited *R. palustris* monocultures
Y_N	5×10^8	N_2-limited *R. palustris* growth yield cells / µmol N_2; N_2-limited *R. palustris* culture[a]
F_c	3.44×10^{-7}	organic acids produced per *E. coli* cell during growth (µmol glucose / cell); Fig 2 time-course: the sum of organic acids left over in coculture (2.20×10^{-7} µmol/ *E.coli* cell) + what was estimated to be assimilated by *R. palustris* (1.24×10^{-7})
F_f	2×10^{-7}	formate produced per *E. coli* cell during growth (µmol glucose / cell); Fig 2 time-course: what was left over in coculture
r_c	300×10^{-11}	*E. coli* growth-independent organic acid production rate (µmol glucose / cell / h); (18)
r_f	50×10^{-11}	*E. coli* growth-independent formate production rate (µmol glucose / cell / h); (18)

Table S5. Extracellular concentrations of nucleobase-containing compounds in CGA0092 and TIE-1 supernatants.

	Concentration (µM / OD)											
	CGA0092						TIE-1					
**Compound**	**Exponential**			**Stationary**			**Exponential**			**Stationary**		
uracil	0.58	±	0.11	0.26	±	0.03	0.54	±	0.06	0.37	±	0.03
hypoxanthine	0.18	±	0.02	0.12	±	0.01	ND			0.03	±	0.01
adenine	17.37	±	1.67	14.36	±	2.25	0.31	±	0.12	0.19	±	0.03
xanthine	0.08	±	0.02	0.07	±	0.04	0.07	±	0.00	0.04	±	0.01
guanine	0.11	±	0.03	0.07	±	0.03	0.09	±	0.02	0.06	±	0.01
thymidine	0.15	±	0.01	0.19	±	0.05	0.22	±	0.01	0.24	±	0.02
uridine	0.15	±	0.05	0.11	±	0.02	0.30	±	0.02	0.34	±	0.05
cytidine	0.21	±	0.07	0.15	±	0.02	0.37	±	0.06	0.45	±	0.07
2'-deoxyinosine	0.06	±	0.02	0.02	±	0.00	0.04	±	0.01	0.02	±	0.00
adenosine	0.52	±	0.20	0.49	±	0.08	0.63	±	0.13	0.56	±	0.05
2'-deoxyguanosine	0.07	±	0.02	0.06	±	0.02	0.06	±	0.01	0.04	±	0.00
inosine	0.13	±	0.02	0.12	±	0.04	0.07	±	0.01	0.05	±	0.00
xanthosine	0.12	±	0.02	0.10	±	0.01	0.13	±	0.01	0.11	±	0.01
guanosine	0.17	±	0.06	0.12	±	0.02	0.17	±	0.03	0.15	±	0.01
TMP	0.09	±	0.03	0.05	±	0.01	0.08	±	0.02	0.07	±	0.01
CMP	0.17	±	0.06	0.10	±	0.01	0.13	±	0.02	0.23	±	0.01
UMP	0.19	±	0.03	0.11	±	0.01	0.21	±	0.03	0.33	±	0.02
c-GMP	0.07	±	0.02	0.03	±	0.01	0.06	±	0.01	0.04	±	0.00
AMP	0.10	±	0.03	0.08	±	0.02	0.13	±	0.02	0.20	±	0.01
GMP	0.08	±		0.06	±	0.00	0.16	±	0.03	0.20	±	0.04
ADP	0.21	±	0.06	0.11	±	0.02	0.21	±	0.04	0.19	±	0.02
GDP	0.12	±		0.07	±	0.02	0.13	±	0.04	0.13	±	0.04
ATP	0.35	±	0.21	0.20	±	0.03	0.43	±	0.07	0.05	±	0.02
GTP	ND			ND			0.09	±		0.05	±	0.01
c-di-GMP	0.08	±	0.03	0.03	±	0.01	0.07	±	0.02	0.04	±	0.01
FAD	0.10	±	0.03	0.04	±	0.00	0.08	±	0.01	0.04	±	0.01

ND, not detected. Values are means ± SD; n= 1-3. Triplicate samples were run in each case but not all compounds were detected in each sample. Values without SD indicate that a compound was only detected in one of the three replicates.

Table S6. Intracellular concentrations of nucleobase-containing compounds in CGA0092 and TIE-1 supernatants.

	Concentration (µM)											
	CGA0092						TIE-1					
Compound	Exponential			Stationary			Exponential			Stationary		
uracil	30.0	±	8.4	19.5	±	4.9	31.2	±	7.1	44.2	±	2.2
hypoxanthine	29.5	±	7.7	24.1	±	5.7		ND			ND	
adenine	1516.8	±	645.0	1467.5	±	554.3	17.0	±	3.1	13.7	±	4.5
xanthine		ND		12.6	±	4.2	11.8	±		7.6	±	
guanine	22.6	±	3.4	20.5	±	3.6	22.9	±	6.3	21.7	±	1.0
thymidine	23.3	±	8.0	26.8	±	3.6	27.8	±	11.0	42.2	±	3.1
uridine	18.4	±	1.8	14.7	±	1.9	28.8	±	7.4	46.8	±	5.1
cytidine	35.9	±	4.0	32.9	±	6.8	53.2	±	14.5	102.0	±	11.8
2'-deoxyinosine	10.8	±	2.4	5.1	±	0.6	9.6	±	1.8	4.7	±	0.1
adenosine	67.1	±	11.7	67.8	±	1.9	59.9	±	25.6	105.9	±	16.3
2'-deoxyguanosine	15.9	±	3.0	14.2	±	4.2	15.1	±	3.1	11.4	±	1.0
inosine	24.6	±	6.1	22.4	±	3.1	16.6	±	3.7	13.2	±	0.3
xanthosine	24.7	±		20.3	±	2.2	22.6	±	5.6	26.4	±	3.3
guanosine	32.9	±	4.4	31.3	±	5.1	32.0	±	9.8	47.5	±	2.3
TMP	25.0	±	8.5	13.8	±	0.7	21.5	±	6.9	17.5	±	2.2
CMP	54.0	±	22.1	27.0	±	5.0	36.4	±	5.7	84.1	±	22.2
UMP	47.4	±	13.0	41.0	±	7.3	53.2	±	10.1	97.3	±	7.4
c-GMP	17.8	±	4.0	10.0	±	0.5	17.5	±	3.7	10.6	±	0.3
AMP	84.3	±	20.3	142.9	±	22.1	105.7	±	24.7	167.6	±	9.5
GMP	40.5	±		33.7	±	6.1	57.1	±	18.6	58.2	±	9.1
ADP	168.6	±	12.6	244.5	±	69.9	177.0	±	3.7	217.9	±	38.1
GDP	65.2	±		54.0	±	8.6	63.3	±	8.2	82.0	±	8.9
ATP	356.8	±	97.0	406.9	±	187.9	301.9	±	49.3	319.1	±	46.7
GTP	59.0	±	11.8	44.6	±	8.4	53.4	±	5.7	81.2	±	19.0
c-di-GMP	21.8	±	1.9	13.5	±	3.3	18.1	±	6.5	12.8	±	1.3
FAD	38.4	±	10.7	29.1	±	8.4	33.6	±	3.7	32.5	±	9.0

ND, not detected. Values are means ± SD; n= 1-3. Triplicate samples were run in each case but not all compounds were detected in each sample. Values without SD indicate that a compound was only detected in one of the three replicates.

Supplementary Figure and Table References:

1. Xiang TX, Anderson BD. 1994. The relationship between permeant size and permeability in lipid bilayer membranes. J Membr Biol 140:111-22.
2. Mazny BE, Sheff OF, LaSarre B, McKinlay A, McKinlay JB. 2023. Complete genome sequence of *Rhodopseudomonas palustris* CGA0092 and corrections to the *R. palustris* CGA009 genome sequence. Microbiol Resour Announc 12:e0128522.
3. LaSarre B, McCully AL, Lennon JT, McKinlay JB. 2017. Microbial mutualism dynamics governed by dose-dependent toxicity of cross-fed nutrients. ISME J 11:337-348.
4. Jiao Y, Kappler A, Croal LR, Newman DK. 2005. Isolation and characterization of a genetically tractable photoautotrophic Fe(II)-oxidizing bacterium, *Rhodopseudomonas palustris* strain TIE-1. Appl Environ Microbiol 71:4487-96.
5. Xing D, Zuo Y, Cheng S, Regan JM, Logan BE. 2008. Electricity generation by *Rhodopseudomonas palustris* DX-1. Environ Sci Technol 42:4146-51.
6. Fixen KR, Oda Y, Harwood CS. 2016. Clades of photosynthetic bacteria belonging to the genus *Rhodopseudomonas* show marked diversity in light-harvesting antenna complex gene composition and expression. mSystems 1:e00006-15.
7. Kamal VS, Wyndham RC. 1990. Anaerobic phototrophic metabolism of 3-chlorobenzoate by *Rhodopseudomonas palustris* WS17. Appl Environ Microbiol 56:3871-3.
8. Jung G, Jung H, Kim J, Ahn Y, Park S. 1999. Isolation and characterization of *Rhodopseudomonas palustris* P4 which utilizes CO with the production of H_2. Biotechnol Lett 21:525-529.
9. Inui M, Roh JH, Zahn K, Yukawa H. 2000. Sequence analysis of the cryptic plasmid pMG101 from *Rhodopseudomonas palustris* and construction of stable cloning vectors. Appl Environ Microbiol 66:54-63.
10. Oda Y, Meijer WG, Gibson JL, Gottschal JC, Forney LJ. 2004. Analysis of diversity among 3-chlorobenzoate-degrading strains of *Rhodopseudomonas palustris*. Microb Ecol 47:68-79.
11. Carlozzi P, Pushparaj B, Degl'Innocenti A, Capperucci A. 2006. Growth characteristics of *Rhodopseudomonas palustris* cultured outdoors, in an underwater tubular photobioreactor, and investigation on photosynthetic efficiency. Appl Microbiol Biotechnol 73:789-95.
12. Oda Y, Larimer FW, Chain PSG, Malfatti S, Shin MV, Vergez LM, Hauser L, Land ML, Braatsch S, Beatty JT, Pelletier DA, Schaefer AL, Harwood CS. 2008. Multiple genome sequences reveal adaptations of a phototrophic bacterium to sediment microenvironments. Proc Natl Acad Sci USA 105:18543-18548.
13. Blattner FR, Plunkett G, 3rd, Bloch CA, Perna NT, Burland V, Riley M, Collado-Vides J, Glasner JD, Rode CK, Mayhew GF, Gregor J, Davis NW, Kirkpatrick HA, Goeden MA, Rose DJ, Mau B, Shao Y. 1997. The complete genome sequence of *Escherichia coli* K-12. Science 277:1453-62.

14. Baba T, Ara T, Hasegawa M, Takai Y, Okumura Y, Baba M, Datsenko KA, Tomita M, Wanner BL, Mori H. 2006. Construction of *Escherichia coli* K-12 in-frame, single-gene knockout mutants: the Keio collection. Mol Syst Biol 2:2006.0008.
15. Datsenko KA, Wanner BL. 2000. One-step inactivation of chromosomal genes in *Escherichia coli* K-12 using PCR products. Proc Natl Acad Sci USA 97:6640-5.
16. Cherepanov PP, Wackernagel W. 1995. Gene disruption in *Escherichia coli*: TcR and KmR cassettes with the option of Flp-catalyzed excision of the antibiotic-resistance determinant. Gene 158:9-14.
17. McKinlay JB, Harwood CS. 2010. Carbon dioxide fixation as a central redox cofactor recycling mechanism in bacteria. Proc Natl Acad Sci USA 107:11669-75.
18. McCully AL, LaSarre B, McKinlay JB. 2017. Growth-independent cross-feeding modifies boundaries for coexistence in a bacterial mutualism. Environ Microbiol 19:3538-3550.
19. Buhr A, Daniels GA, Erni B. 1992. The glucose transporter of *Escherichia coli*. Mutants with impaired translocation activity that retain phosphorylation activity. J Biol Chem 267:3847-51.
20. Papakostas K, Botou M, Frillingos S. 2013. Functional identification of the hypoxanthine/guanine transporters YjcD and YgfQ and the adenine transporters PurP and YicO of *Escherichia coli* K-12. J Biol Chem 288:36827-40.
21. LaSarre B, Kysela DT, Stein BD, Ducret A, Brun YV, McKinlay JB. 2018. Restricted localization of photosynthetic intracytoplasmic membranes (ICMs) in multiple genera of purple nonsulfur bacteria. mBio 9:e00780-18.
